# The fundamental role of mitochondria–endoplasmic reticulum contacts in ageing and declining healthspan

**DOI:** 10.1098/rsob.240287

**Published:** 2025-02-12

**Authors:** Richard M. Monaghan

**Affiliations:** ^1^ British Heart Foundation Centre of Research Excellence Manchester, Division of Cardiovascular Sciences, School of Medical Sciences, Faculty of Biology, Medicine, and Health, The University of Manchester, The AV Hill Building, Manchester M13 9PT, UK

**Keywords:** mitochondria, endoplasmic reticulum, ageing, membrane contact sites, healthspan, metabolism

## Abstract

This open question research article highlights mitochondria-associated endoplasmic reticulum (ER) membranes (MAMs), which have emerged as crucial cellular structures that challenge our traditional understanding of organelle function. This review highlights the critical importance of MAMs as a frontier in cell biology with far-reaching implications for health, disease and ageing. MAMs serve as dynamic communication hubs between the ER and mitochondria, orchestrating essential processes such as calcium signalling, lipid metabolism and cellular stress responses. Recent research has implicated MAM dysfunction in a wide array of conditions, including neurodegenerative diseases, metabolic disorders, cardiovascular diseases and cancer. The significant lack of biological knowledge behind MAM function emphasizes the need to study these enigmatic subcellular sites in greater detail. Key open questions include the mechanisms controlling MAM formation and disassembly, the full complement of MAM-associated proteins and how MAMs contribute to cellular decision-making and ageing processes. Advancing our understanding of MAMs through interdisciplinary approaches and cutting-edge technologies promises to reveal new insights into fundamental cellular signalling pathways and potentially lead to innovative therapeutic strategies for a range of diseases. As such, MAM research represents a critical open question in biology with the potential to transform our understanding of cellular life and human health.

**Figure:**
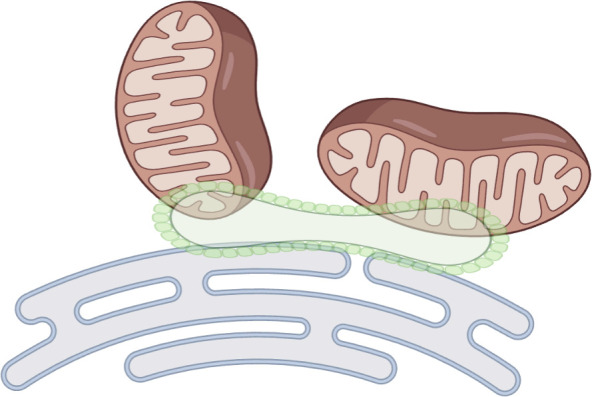

## 1. Introduction: rethinking cellular organization

For decades, scientists viewed the various compartments within cells, called organelles, as relatively independent entities. This perspective, while useful for understanding basic cellular structure, has proven to be an oversimplification of the complex and dynamic nature of cellular organization. Recent research has revealed a far more interconnected and fluid cellular landscape, where organelles interact and communicate in sophisticated ways [1,2]. This paradigm shift has led to a new understanding of cellular function, with implications for our comprehension of both normal physiology and disease states.

At the heart of this paradigm shift is the discovery of specialized regions where two critical organelles—mitochondria and the endoplasmic reticulum (ER)—are in close apposition. These regions, known as mitochondria-associated ER membranes (MAMs), are revolutionizing our understanding of cellular function and disease [3,4]. First identified several decades ago through biochemical fractionation studies, MAMs have since been recognized as dynamic structures playing pivotal roles in various cellular processes [5].

MAMs act as cellular ‘communication hubs’, allowing for rapid and precise exchange of signals and molecules between mitochondria and the ER. This communication is crucial for maintaining cellular health, responding to stress and regulating energy production. The strategic positioning of MAMs allows for efficient transfer of molecules and signals, facilitating precise control of cellular functions [6,7]. MAMs actively adapt to cellular needs, dynamically modifying their composition and function in response to stimuli [8].

Recent advancements in proteomic analyses have revealed that MAMs contain over 1000 enriched proteins across different species and tissues, highlighting their complex and diverse functions [9,10]. This protein composition is not static but rather dynamic, responding to cellular needs and environmental cues. The intricate network of proteins at MAMs orchestrates a myriad of cellular functions, making these contact sites central to cellular homeostasis and adaptation to stress [11,12].

As our understanding of MAMs has grown, so too has the recognition of their importance in health and disease. Alterations in MAM structure and function have been implicated in a wide range of conditions, including neurodegenerative diseases, metabolic disorders and cardiovascular disease [13–15]. Furthermore, the role of MAMs in cellular ageing processes has garnered significant attention, suggesting that these structures may be key players in systemic ageing [16,17].

## 2. The multifaceted functions of MAMs

**Figure 1.**
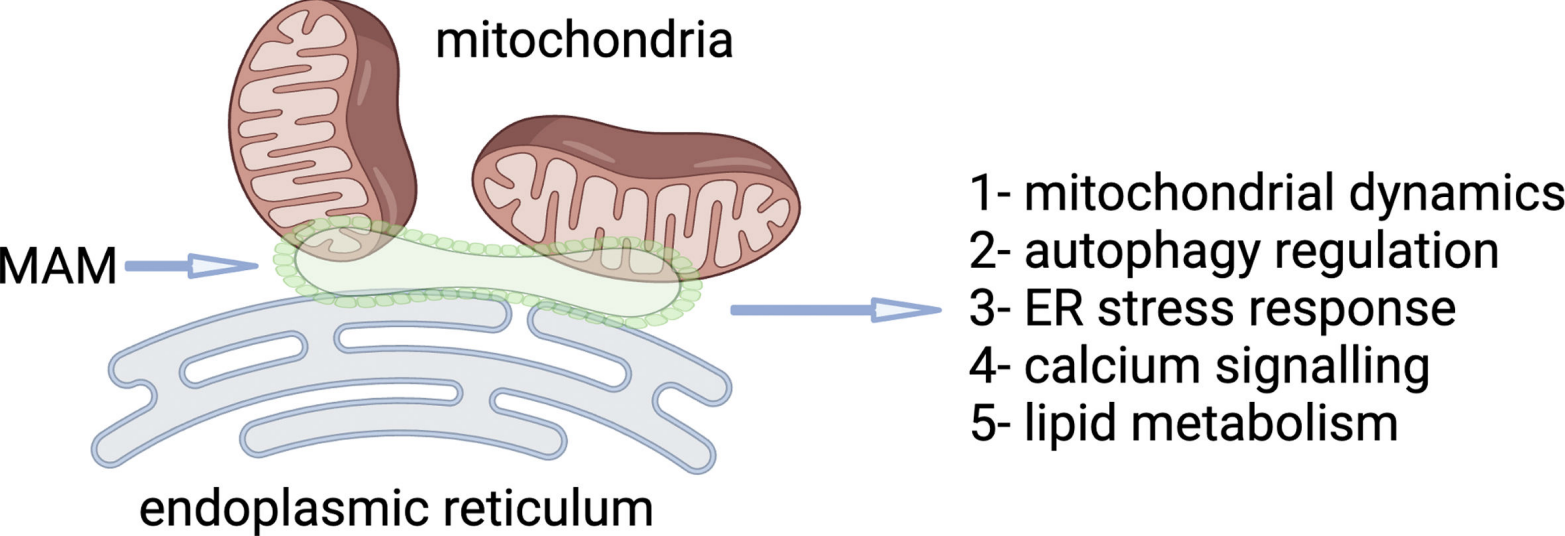
Functions of mitochondria-associated ER membranes (MAMs). Schematic representation of the diverse roles of MAMs in cellular regulation. Key functions include calcium signalling, lipid metabolism, mitochondrial dynamics, autophagy regulation, ER stress response, innate immune signalling, apoptosis regulation and metabolic control. Each function is highlighted with its cellular context, emphasizing MAMs as hubs of organelle communication. Understanding these processes reveals insights into MAMs’ critical roles in health, ageing and disease. Image generated using BioRender.

Before exploring the open questions in MAM research, it is crucial to understand the diverse functions these structures play in cellular biology (figure 1).

*1. Calcium signalling*. MAMs play a critical role in regulating calcium transfer between the ER and mitochondria. This process is essential for energy production, cell survival and various signalling pathways [18,19]. Recent studies have shown that MAMs can act as calcium signalling microdomains, allowing for precise spatial and temporal control of calcium-dependent processes [20].

*2. Lipid metabolism*. MAMs are sites of lipid synthesis and transfer, crucial for maintaining the unique lipid composition of mitochondrial membranes [21]. New research has revealed that MAMs are also involved in the regulation of lipid droplet formation and the transfer of lipids between organelles, mediated by lipid transport proteins, regulating the amount of specific lipid classes in subcellular membranes and highlighting their importance in cellular lipid homeostasis [22,23].

*3. Mitochondrial dynamics*. These contact sites are involved in regulating mitochondrial fission and fusion, processes that are essential for maintaining mitochondrial health [24,25]. Recent studies have shown that MAMs serve as platforms for the assembly of the mitochondrial fission machinery, underscoring their importance in mitochondrial quality control [26].

*4. ER stress response.* MAMs are implicated in the cellular response to ER stress, playing a role in the unfolded protein response (UPR) [27,28]. New research has revealed that MAMs can modulate the activity of key UPR sensors, thereby influencing cellular fate decisions under stress conditions [29].

*5. Autophagy regulation*. Emerging evidence suggests that MAMs are involved in the initiation of autophagy, a critical cellular quality control mechanism [30,31]. Recent studies have shown that MAMs serve as sites for the formation of autophagosomes, the double-membrane vesicles that engulf cellular material for degradation [32].

*6. Innate immune signalling*. A newly discovered function of MAMs is their role in innate immune signalling. Recent studies have shown that MAMs act as staging areas for the formation of inflammasomes—groups of proteins that trigger the body’s inflammatory response when assembled [33,34].

*7. Apoptosis regulation:* MAMs play a crucial role in the regulation of apoptosis or programmed cell death. The transfer of calcium from the ER to mitochondria via MAMs can trigger apoptosis under certain conditions, while MAM-mediated signalling can also promote cell survival [35,36].

*8. Metabolic regulation:* Recent studies have highlighted the importance of MAMs in metabolic regulation. MAMs have been shown to play a role in insulin signalling and glucose homeostasis, with implications for metabolic disorders such as diabetes [37,38].

Understanding these diverse functions provides context for the open questions that follow and highlights the far-reaching impact of MAM biology on cellular health and disease.

## 3. Key open questions in MAM research

### 3.1. How do cells regulate MAM formation and disassembly?

While we know that MAMs are dynamic structures, the precise mechanisms controlling their formation and breakdown remain unclear. Studies have identified several proteins involved in tethering the ER to mitochondria, such as Mitofusin 2 and the VAPB-PTPIP51 complex [39,40]. However, the signalling pathways that regulate these tethers and how cells adjust MAM formation in response to different stimuli are not fully understood.

Recent research has begun to shed light on this question. For instance, a 2020 study by Toyofuku *et al*. identified a novel regulatory mechanism involving the Parkinson’s disease-associated protein LRRK2, which modulates ER–mitochondria tethering through phosphorylation of Rab32 [41]. MOSPD2 plays a critical role in regulating MAMs and ER–lipid droplet contact sites, shedding light on their mechanistic control [42]. A key role for MIGA2 in mediating lipid transport between mitochondrial and ER membranes, where bespoke FFAT motifs in MOSPD2 that harbour serine/threonine residues to allow for regulatory upstream phosphorylation has been previously observed [43,44].

However, many questions remain. How do cells sense the need for increased or decreased MAM formation? What are the molecular switches that trigger MAM assembly or disassembly? How is the specificity of MAM formation at particular sites on the ER and mitochondrial membranes determined? Answering these questions could provide new ways to modulate cellular function in health and disease.

### 3.2. What is the full complement of proteins involved in MAM function?

Recent studies have identified over 1000 proteins enriched at MAMs, but the roles of many of these proteins are still unknown [9,10]. Some key players, such as the IP3R-Grp75-VDAC complex involved in calcium transfer, have been well-characterized [45]. However, the functions of many other MAM-associated proteins remain to be elucidated.

Recent proteomic studies have continued to expand our understanding of the MAM proteome. For example, a 2022 study by Wilson *et al*. used proximity labelling techniques to identify novel MAM-associated proteins in different tissues, revealing tissue-specific differences in MAM composition [46]. Another study by Obara *et al*. applied super-resolution microscopy combined with proteomics to map the nanoscale organization of proteins at MAMs, revealing distinct subdomains within these contact sites [47].

Despite these advances, many questions remain. What are the functions of the numerous uncharacterized proteins found at MAMs? How does the composition of MAMs change in response to different cellular states or stresses? Are there disease-specific alterations in the MAM proteome that could serve as biomarkers or therapeutic targets? Deciphering this complex protein network is crucial for understanding MAM function and identifying potential therapeutic targets.

### 3.3. How do MAMs contribute to cellular decision-making?

MAMs appear to be involved in determining cell fate, including decisions between survival and death. For instance, excessive calcium transfer from the ER to mitochondria via MAMs can trigger apoptosis [46]. Conversely, MAM-mediated signalling can also promote cell survival under certain conditions [47]. The mechanisms governing these cellular ‘decisions’ remain unclear and represent a promising area for future research.

Recent studies have begun to unravel the complex signalling networks at MAMs that influence cellular decision-making. For example, a 2023 study by Zhang *et al*. revealed that MAMs serve as signalling hubs for the integration of metabolic and stress signals, influencing cellular decisions about growth, survival and death [48]. Another study by Ziegler *et al*. in 2021 showed that MAMs play a crucial role in determining cellular senescence, a state of permanent cell cycle arrest associated with ageing and various diseases [49].

However, many questions remain unanswered. How do cells integrate the various signals received at MAMs to make coherent decisions? What are the molecular mechanisms that allow MAMs to switch between pro-survival and pro-death signalling? How do these decision-making processes at MAMs contribute to tissue homeostasis and disease pathogenesis? Understanding these mechanisms could provide new insights into cellular behaviour in health and disease.

### 3.4. How do MAMs change during ageing and how does this impact cellular health?

While some age-related changes in MAMs have been observed, such as alterations in calcium signalling and mitochondrial function [50], the full impact of these changes on cellular function and organismal health remains an open question. Understanding how MAMs change throughout the lifespan could provide insights into the ageing process and potentially lead to interventions to promote healthy ageing.

Disruption of MAMs impairs the structural and functional connectivity between the ER and mitochondria, leading to significant cellular dysfunction. Morphological defects include altered organelle juxtaposition and compromised membrane integrity, which impede critical processes such as calcium homeostasis, lipid metabolism and mitochondrial dynamics [51]. For instance, studies have shown that high glucose levels can disrupt MAM integrity through the pentose phosphate pathway, resulting in mitochondrial fragmentation and altered respiration. Additionally, mutations in proteins like FUS have been linked to decreased MAM formation, leading to mitochondrial dysfunctions in oligodendroglial progenitor cells [52]. These disruptions are associated with various pathologies, including neurodegenerative diseases and metabolic disorders, underscoring the critical role of MAM integrity in maintaining cellular homeostasis.

Recent studies have begun to explore the relationship between MAMs and ageing. For instance, a 2018 study by Janikiewicz *et al*. found that ageing is associated with a decrease in MAM integrity and function, leading to impaired mitochondrial calcium uptake and reduced cellular bioenergetics [53]. Another study by Calvo-Rodriguez *et al*. in 2021 revealed that age-related changes in MAM composition contribute to increased neuronal vulnerability to stress in Alzheimer’s disease [54].

However, many questions remain. How do age-related changes in MAM function contribute to the decline in cellular and organismal health? Are there specific MAM-associated proteins or lipids that could serve as biomarkers of cellular ageing? Could interventions targeting MAMs help to slow or reverse age-related cellular dysfunction? Answering these questions could lead to new strategies for promoting healthy ageing and treating age-related diseases.

### 3.5. How can we target MAMs therapeutically?

Given their importance in cellular function, MAMs represent a promising target for new therapies. However, developing drugs that specifically modulate MAM function without disrupting other cellular processes remains a significant challenge. Some potential approaches include targeting MAM-specific proteins or developing compounds that alter MAM formation or stability [55]. Further research is needed to identify the most effective and safe ways to therapeutically modulate MAM function.

Recent studies have begun to explore potential therapeutic approaches targeting MAMs. Zhang *et al*. highlight the therapeutic potential of a small molecule enhancer of mitochondria–ER coupling in models of neurodegeneration [48], yet the specific proteins targeted by this molecule remain an area of interest. Exploring these proteins could provide deeper insights into the molecular mechanisms underlying improved mitochondria–ER communication and their downstream effects on cellular health, particularly in the context of neurodegenerative diseases. Understanding these targets may also open avenues for the development of more precise therapeutic strategies [56]. Another study demonstrated that modulation of MAM-associated calcium channels could protect against cardiac ischaemia-reperfusion injury [57].

However, many challenges remain. How can we achieve specificity in targeting MAMs without affecting other cellular processes? What are the potential long-term effects of modulating the MAM function? How can we develop therapies that target MAMs in specific tissues or cell types? Addressing these questions will be crucial for translating our understanding of MAM biology into effective therapies.

## 4. Potential implications and importance

The study of MAMs has far-reaching implications for our understanding of cellular biology and human health.

*1. Cardiovascular health.* MAMs play a crucial role in calcium signalling and energy production in heart cells. Disruptions in MAM function have been linked to heart failure and arrhythmias [58,59]. For example, alterations in the IP3R-Grp75-VDAC complex at MAMs can lead to calcium overload in mitochondria, triggering cardiac dysfunction [60]. Recent studies have also implicated MAMs in the pathogenesis of atherosclerosis, with alterations in MAM function contributing to endothelial dysfunction and plaque formation [61].

In heart failure, MAM dysfunction leads to dysregulated calcium transfer, mitochondrial calcium overload, excessive ROS production and aberrant RyR2-mediated calcium release, causing impaired cardiac function [59]. Targeting MAM integrity can help restore calcium homeostasis, suggesting therapeutic potential for MAM-focused interventions [58,59].

*2. Neurodegenerative diseases.* Alterations in MAM function have been observed in conditions like Alzheimer’s and Parkinson’s disease [62,63]. In Alzheimer’s disease, the presenilin proteins involved in amyloid-β production are enriched at MAMs, and disruptions in MAM-mediated calcium signalling may contribute to neuronal death [64]. Recent studies have also implicated MAM dysfunction in the pathogenesis of amyotrophic lateral sclerosis (ALS) and frontotemporal dementia, highlighting the broad relevance of MAMs to neurodegenerative disorders [65].

In Alzheimer’s disease, upregulated MAM function [64] leads to mitochondrial calcium overload, excessive ROS production and impaired lipid metabolism, impacting Aβ production and aggregation [65]. Aβ accumulation at MAMs exacerbates calcium dysregulation, leading to synaptic dysfunction and neuronal death. Targeting MAM function shows promise in Alzheimer’s disease models [63,64].

*3. Metabolic disorders*. MAMs are involved in lipid metabolism and insulin signalling. Dysfunction in these areas has been implicated in conditions like diabetes and obesity [51,66]. For instance, alterations in MAM-mediated lipid transfer can affect insulin sensitivity in liver cells [67]. Recent studies have also revealed that MAM dysfunction contributes to the development of non-alcoholic fatty liver disease, a growing health concern worldwide [68,69]. Understanding the role of MAMs in metabolic regulation could lead to new approaches for treating metabolic disorders.

*4. Cancer*. Growing evidence indicates that cancer cells might change how MAMs work to help them grow and survive [70]. For instance, some cancer cells seem to adjust calcium signalling at MAMs to avoid programmed cell death and encourage rapid multiplication [71]. Recent studies have also implicated MAMs in cancer metastasis, with alterations in MAM function contributing to changes in cell migration and invasion [72].

In cancer, increased ER–mitochondria connections [71] lead to higher calcium transfer, promoting cell survival, ATP production and altered lipid metabolism crucial for metastasis [72]. Some MAM-associated proteins also inhibit apoptosis [70]. Certain chemotherapeutics disrupt MAM integrity, suggesting MAM-targeted therapies’ potential in cancer treatment [73]. Understanding these changes could lead to new therapies.

*5. Ageing*. Changes in MAM function may contribute to the cellular decline associated with ageing [17,74]. Age-related alterations in MAM structure and function have been observed in various tissues, potentially contributing to the decline in mitochondrial function and increased oxidative stress seen in ageing cells [73]. Recent studies have also implicated MAM dysfunction in age-related decline in stem cell function, suggesting a broader role for MAMs in tissue regeneration and homeostasis [75]. Interventions targeting MAMs could potentially promote healthier ageing by maintaining cellular homeostasis and energy production.

*6. Infectious diseases*. Recent research has uncovered an important role for MAMs in the cellular response to viral infections. MAMs have been shown to serve as platforms for innate immune signalling and the replication of certain viruses [76,77]. For example, a 2022 study revealed that SARS-CoV-2, the virus responsible for COVID-19, manipulates MAMs to promote its replication [78]. Understanding the role of MAMs in viral infections could lead to new antiviral strategies.

*7. Autoimmune disorders*. Emerging evidence suggests that MAM dysfunction may contribute to the pathogenesis of certain autoimmune disorders. For instance, recent studies have implicated MAM alterations in systemic lupus erythematosus and rheumatoid arthritis [79,80]. Understanding the role of MAMs in autoimmune processes could lead to new therapeutic approaches for these challenging conditions.

*8. Developmental biology*. MAMs play crucial roles in various aspects of cellular and organismal development. Recent studies have revealed the importance of MAMs in processes such as embryonic stem cell differentiation, neuronal development and tissue morphogenesis [81,82]. Understanding the developmental roles of MAMs could provide insights into congenital disorders and regenerative medicine approaches.

## 5. Future directions

**Figure 2.**
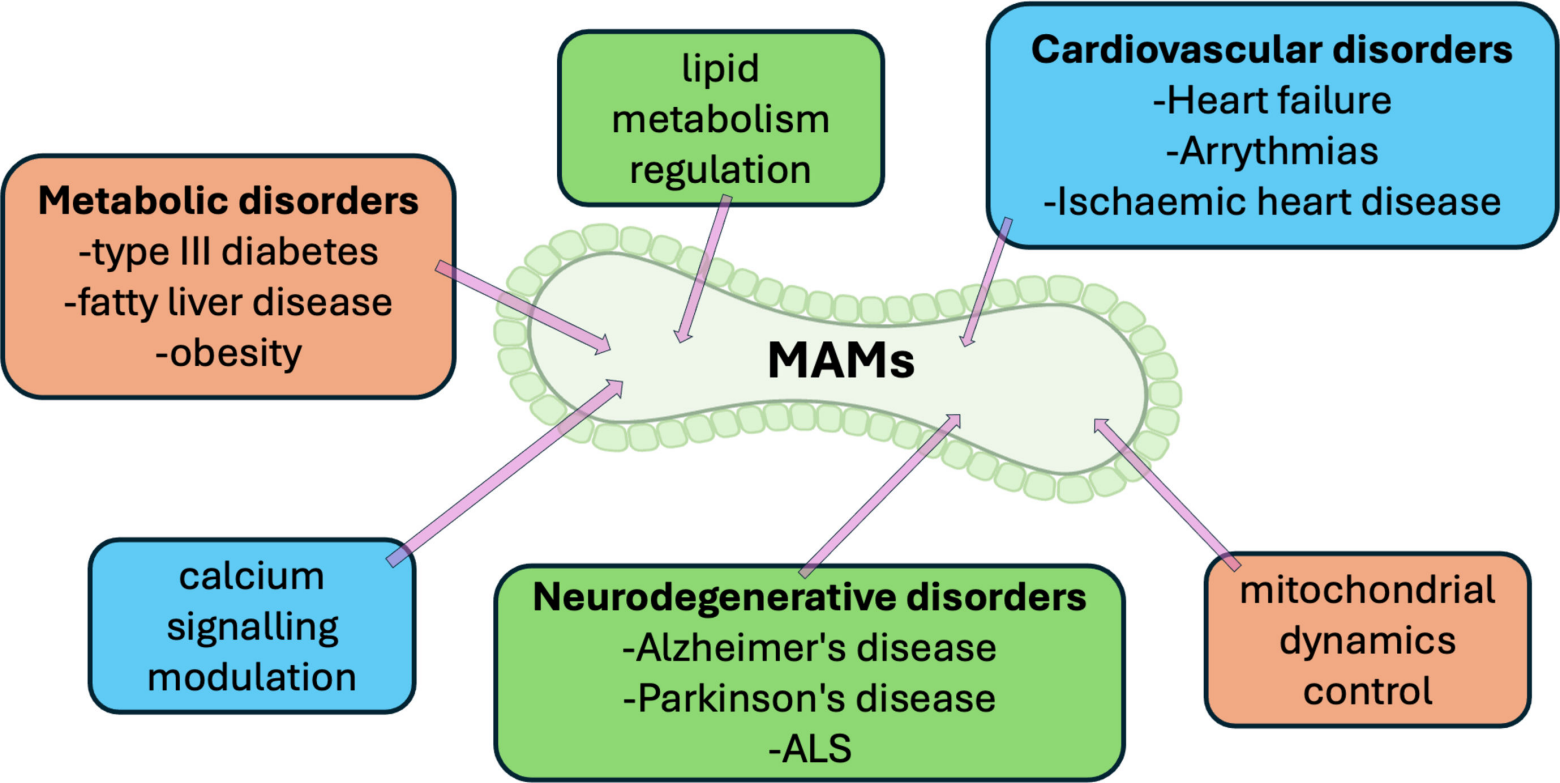
Therapeutic approaches for targeting MAM-associated disorders. Overview of potential therapeutic strategies targeting mitochondria-associated ER membranes (MAMs) to address diseases like neurodegenerative disorders, cardiovascular conditions, metabolic diseases and cancer. The diagram illustrates intervention pathways, such as modulating calcium signalling, enhancing MAM integrity and targeting specific MAM proteins or lipid transfer mechanisms. Future research directions include nanoparticle-based drug delivery and optogenetic tools to manipulate MAM functionality. See the main text and electronic supplementary material, table S1 for references.

The field of MAM research is rapidly evolving (see figure 2 and table 1 for therapeutic approaches to targeting MAM-associated disorders; see electronic supplementary material, table S1 for a comprehensive list of contact site proteins with references and details of their function, if known, in MAMs that could be potential therapeutic targets), with several exciting directions for future study:

*1. Advanced imaging techniques*. Developing new methods to visualize MAMs in living cells will be crucial for understanding their dynamics and function. Advanced imaging methods, like super-resolution microscopy, can now show us MAMs in extreme detail, down to billionths of a metre [83]. Another technique, cryoelectron tomography, offers the potential to reveal the three-dimensional structure of MAMs at a very small scale [84]. Recent advances in live-cell imaging techniques, such as lattice light-sheet microscopy, are enabling researchers to observe MAM dynamics in real time with unprecedented spatial and temporal resolution [85]. These advanced imaging approaches will provide unprecedented insights into MAM structure and dynamics, potentially revealing new aspects of their function and regulation.

**Table 1.** Therapeutic approaches to targeting MAMs. Summary of diseases linked to MAM dysfunction and their therapeutic targets. Includes a brief description of associated pathologies (e.g. Alzheimer’s disease, diabetes and cardiovascular disorders) and proposed mechanisms for intervention. Key targets, such as calcium channels, lipid transfer proteins and autophagy regulators, are detailed. This table highlights the translational potential of MAM-focused research for clinical applications.

disease and area	current understanding	therapeutic approach	key challenges	status
neurodegenerative disease	MAM dysfunction leads to calcium overload, protein aggregation, and neuronal death; particularly important in Alzheimer's where presenilin proteins accumulate at MAMs	— small molecule enhancers of ER–mitochondria coupling — targeting calcium transfer mechanisms — modulating protein aggregation at MAMs	— blood-brain barrier penetration — cell-type specific targeting — long-term safety concerns	pre-clinical studies showing promise for small molecule enhancers
cardiovascular disease	MAM dysfunction causes dysregulated calcium transfer, ROS production, and impaired cardiac function; IP3R-Grp75-VDAC complex crucial for heart health	— modulation of MAM-associated calcium channels — targeting RyR2-mediated calcium release — stabilizing MAM integrity	— Cardiac-specific delivery — maintaining normal calcium homeostasis — avoiding arrhythmogenic effects	early clinical trials for calcium channel modulators
metabolic disorders	MAMs regulate lipid metabolism and insulin signalling; dysfunction linked to diabetes, obesity and fatty liver disease	— enhancing MAM-mediated lipid transfer — improving insulin sensitivity — targeting lipid metabolism regulation	— multiple tissue involvement — complex metabolic networks — systemic effects	pre-clinical development of lipid transfer modulators
cancer	cancer cells alter MAM function to avoid cell death and promote growth; enhanced ER-mitochondria connections support metastasis	— targeting calcium-dependent cell death pathways — disrupting cancer-specific MAM alterations — combination with chemotherapy	— cancer cell adaptation — selective targeting — resistance development	early-stage trials in combination therapy
infectious disease	viruses (including SARS-CoV-2) manipulate MAMs for replication; MAMs serve as platforms for innate immune response	— disrupting virus–MAM interactions — enhancing innate immune signalling — targeting viral proteins at MAMs	— viral mutation — maintaining host cell function — specificity of targeting	pre-clinical research stage
age-related conditions	age-related MAM dysfunction affects calcium homeostasis and mitochondrial function; Links to cellular senescence	— maintaining MAM integrity — supporting calcium homeostasis — reducing oxidative stress	— multiple tissue effects — complex age-related changes — long-term intervention needs	experimental stage

*2. Proteomics and interactomics*. Continued refinement of proteomic approaches will provide a more comprehensive understanding of the MAM proteome and how it changes under different conditions. Techniques like proximity labelling (e.g. BioID or APEX) can identify proteins in close spatial proximity within MAMs [86]. Cross-linking mass spectrometry can reveal protein–protein interactions within these complex structures [87]. Recent developments in single-cell proteomics are opening up new possibilities for understanding MAM heterogeneity within and between cell populations [88]. These approaches will help map the intricate protein networks that govern MAM function and how they change in response to cellular state and environmental cues.

*3. Targeted modulation of MAMs*. Developing tools for specific manipulation of MAM structure and function will be crucial for understanding their roles in health and disease. This could include optogenetic tools that allow for light-controlled modulation of MAM formation or disassembly [89]. Engineered proteins that can alter MAM structure or function in response to specific stimuli could provide valuable insights into MAM biology [90]. Recent advances in genome editing techniques, such as CRISPR-Cas9, are enabling more precise manipulation of MAM-associated genes, allowing for detailed functional studies [91].

*4. Therapeutic targeting*. As our understanding of MAMs grows, so too does the potential for developing therapies that target these structures. This could lead to new treatments for cardiovascular diseases, neurodegenerative disorders and age-related conditions. Potential approaches include developing small molecules that modulate specific MAM-associated proteins or using nanoparticles for targeted drug delivery to MAMs [92]. Detailed structural analysis of the lipid transfer protein ERMES at MAMs, using a combination of quantitative live imaging, cryo-correlative microscopy, sub-tomogram averaging and molecular modelling, has greatly enhanced our understanding of lipid transfer mechanisms at MAMs [88].

*5. Systems biology approaches*. Integrating MAM research with broader cellular and organismal physiology will provide a more holistic understanding of their role in health and disease. This could involve combining data from various comprehensive biological approaches—studying genes (genomics), proteins (proteomics) and metabolic processes (metabolomics)—to create detailed models of how MAMs function [93]. Machine learning algorithms could be employed to identify patterns in these complex datasets and predict MAM-related disease outcomes [94]. Recent advances in single-cell multiomics technologies are providing unprecedented insights into cellular heterogeneity and could reveal new aspects of MAM function in diverse cell types and states [95,96].

*6. In vivo studies*. While much of our understanding of MAMs comes from *in vitro* studies, there is a growing need for *in vivo* research to understand their function in the context of whole organisms. Recent developments in intravital microscopy techniques are enabling researchers to observe MAM dynamics in living animals [97]. Scientists are creating animals with fluorescently tagged MAM proteins, allowing them to observe how MAMs work in living organisms during development and disease [98].

*7. Translational research*. Bridging the gap between basic MAM research and clinical applications will be crucial for realizing the therapeutic potential of this field. This could involve developing MAM-based biomarkers for early disease detection or prognosis [99]. Clinical studies investigating the efficacy of MAM-targeted therapies in human patients will be essential for translating laboratory findings into real-world treatments [100].

*8. Interdisciplinary approaches*. The complexity of MAM biology necessitates collaboration across various scientific disciplines. Combining insights from cell biology, biophysics, chemistry and computational science could lead to breakthroughs in our understanding of MAM function and regulation [101]. For example, recent collaborations between cell biologists and physicists have led to new insights into the biophysical properties of MAMs and how they influence cellular function [102].

## 6. Conclusion: emerging horizons for cell biologists

The study of mitochondria–ER membrane contacts represents an exciting frontier in cell biology with promising implications for health, disease and ageing. As research progresses, we are gaining valuable insights that could lead to innovative therapeutic approaches for a range of conditions.

The dysfunction of MAMs underpins critical pathological processes in ageing and chronic diseases, including neurodegenerative disorders, cardiovascular conditions and metabolic syndromes. These diseases represent a significant global health challenge, with an estimated 55 million people currently living with dementia worldwide and the prevalence expected to double by 2050 [103]. Similarly, metabolic disorders such as type 2 diabetes affect over 450 million people globally, placing immense pressure on healthcare systems. Understanding and targeting MAM dysfunction holds great promise for alleviating these escalating healthcare challenges [104].

MAM biology is a fascinatingly complex field, encompassing crucial roles in calcium signalling, lipid metabolism and cellular stress responses. Continued research using cutting-edge technologies and interdisciplinary methods is likely to yield significant advances in our understanding of these intricate structures [105]. This review underscores the urgency of prioritizing MAM research within the broader context of cellular biology and therapeutic innovation. The scientific community is encouraged to intensify efforts in this promising field, harnessing the latest advances in imaging, molecular biology and bioinformatics. Funding organizations should recognize the transformative potential of MAM research and allocate resources to interdisciplinary projects that could redefine our understanding of ageing and disease. By working collaboratively, researchers can unravel the intricacies of MAM biology and translate these findings into impactful clinical solutions.

MAM research has shown potential relevance to several important health issues, including neurodegenerative, cardiovascular and metabolic diseases, as well as cancer. A deeper grasp of MAM biology could open up new avenues for therapeutic intervention, potentially leading to more effective treatments for challenging medical conditions.

Moreover, studying MAMs provides a unique lens through which to examine cellular ageing processes. Understanding age-related changes in MAM function might contribute to promising strategies for promoting healthy ageing and extending healthspan.

As research in this field advances, it is crucial to maintain scientific rigour and ethical considerations. The complexity of MAM biology underscores the interconnected nature of cellular processes and highlights the importance of holistic approaches to understanding and treating disease.

In conclusion, MAM research represents a key area in biology with far-reaching implications. Continued exploration of these fascinating cellular structures is enhancing our understanding of fundamental biological processes and could contribute to significant advancements in medical treatments, offering hope for improved health outcomes in the future.

## Data Availability

This article has no additional data.
